# The energy sensor AMPK orchestrates metabolic and translational adaptation in expanding T helper cells

**DOI:** 10.1096/fj.202001763RR

**Published:** 2021-03-14

**Authors:** Katharina A. Mayer, Ursula Smole, Ci Zhu, Sophia Derdak, Anastasia A. Minervina, Maria Salnikova, Nadine Witzeneder, Anna Christamentl, Nicole Boucheron, Petra Waidhofer‐Söllner, Michael Trauner, Gregor Hoermann, Klaus G. Schmetterer, Ilgar Z. Mamedov, Martin Bilban, Wilfried Ellmeier, Winfried F. Pickl, Guido A. Gualdoni, Gerhard J. Zlabinger

**Affiliations:** ^1^ Institute of Immunology Center of Pathophysiology, Infectiology and Immunology Medical University of Vienna Vienna Austria; ^2^ Hans Popper Laboratory of Molecular Hepatology Division of Gastroenterology and Hepatology Department of Internal Medicine III Medical University of Vienna Vienna Austria; ^3^ Core Facilities Medical University of Vienna Vienna Austria; ^4^ Department of Genomics of Adaptive Immunity Shemyakin‐Ovchinnikov Institute of Bioorganic Chemistry Moscow Russia; ^5^ Department of Laboratory Medicine Medical University of Vienna Vienna Austria; ^6^ Division of Hematology and Hemostaseology Department of Internal Medicine I Medical University of Vienna Vienna Austria; ^7^ MLL Munich Leukemia Laboratory Munich Germany; ^8^ Central European Institute of Technology Masaryk University Brno Czech Republic; ^9^ Division of Nephrology and Dialysis Department of Internal Medicine III Medical University of Vienna Vienna Austria

**Keywords:** AMPK, cellular metabolism, T cell, translation

## Abstract

The importance of cellular metabolic adaptation in inducing robust T cell responses is well established. However, the mechanism by which T cells link information regarding nutrient supply to clonal expansion and effector function is still enigmatic. Herein, we report that the metabolic sensor adenosine monophosphate‐activated protein kinase (AMPK) is a critical link between cellular energy demand and translational activity and, thus, orchestrates optimal expansion of T cells in vivo. AMPK deficiency did not affect T cell fate decision, activation, or T effector cell generation; however, the magnitude of T cell responses in murine in vivo models of T cell activation was markedly reduced. This impairment was global, as all T helper cell subsets were similarly sensitive to loss of AMPK which resulted in reduced T cell accumulation in peripheral organs and reduced disease severity in pathophysiologically as diverse models as T cell transfer colitis and allergic airway inflammation. T cell receptor repertoire analysis confirmed similar clonotype frequencies in different lymphoid organs, thereby supporting the concept of a quantitative impairment in clonal expansion rather than a skewed qualitative immune response. In line with these findings, in‐depth metabolic analysis revealed a decrease in T cell oxidative metabolism, and gene set enrichment analysis indicated a major reduction in ribosomal biogenesis and mRNA translation in AMPK‐deficient T cells. We, thus, provide evidence that through its interference with these delicate processes, AMPK orchestrates the quantitative, but not the qualitative, manifestation of primary T cell responses in vivo.

**Abbreviations**
AA: antimycin A
ACC: acetyl‐CoA‐carboxylase
AMP: adenosine monophosphate
AMPK: AMP‐activated protein kinase
ATP: adenosine triphosphate
CaMKKβ: calcium/calmodulin‐dependent protein kinase β
ECAR: extracellular acidification rate
FCCP: fluoro‐carbonyl cyanide phenylhydrazone
Foxp3: Forkhead box protein p3
GSEA: gene set enrichment analysis
HDM: house‐dust mite
IFN‐γ: interferon‐γ
IL: interleukin
ILC2: innate lymphoid cell type 2
LC‐MS: liquid chromatography‐mass spectrometry
LCMV: lymphocytic choriomeningitis virus
LKB1: liver kinase B1
mLN: mesenteric lymph node
mTOR: mammalian target of rapamycin
mTORC1: mammalian target of rapamycin complex 1
NF‐κB: nuclear factor kappa‐light‐chain‐enhancer of activated B cells
OCR: oxygen consumption rate
Raptor: regulatory‐associated protein of mTOR
Rot: rotenone
TAK1: TGF‐β‐activated kinase‐1
TCA cycle: tricarboxylic acid cycle
TCR: T cell receptor
TGF‐β: transforming growth factor β
Th: T helper cell
Th1: type 1 T helper cell
Th2: type 2 T helper cell
Th17: type 17 T helper cell
Treg: regulatory T cell
TSC2: tuberous sclerosis complex 2

## 1 INTRODUCTION

Naive CD4 + T cells actively maintain a quiescent state with low metabolic activity. Upon antigen receptor stimulation and receiving co‐stimulatory signals, they exit quiescence and undergo clonal proliferation and effector cell differentiation. These processes require T cell metabolic reprograming to establish an adequate immune response in terms of quality (T helper cell differentiation, activation, and clonal diversity) and quantity (clone size). To comply with this high metabolic demand for rapid growth and biosynthesis, including translation of target genes, activated T cells preferentially increase glycolysis and glutaminolysis, and also rely on proper mitochondrial biogenesis and oxidative phosphorylation.^1^, ^2^, ^3^

The nature of extracellular nutrients may shape T cell responses in vivo, and glucose,^4^, ^5^, ^6^ amino acid^7^, ^8^, ^9^, ^10^, ^11^ and/or fatty acid metabolism^4^, ^12^, ^13^, ^14^ are required to induce the appropriate T cell response upon antigenic stimulation. Therefore, T cells rely on the expression of nutrient transporters^5^, ^15^ and on the activity of hallmark metabolic regulators that orchestrate the rewiring of cellular intermediate metabolism and integrate information on the provision of nutrients to translational control of T cell expansion. Consequently, manipulation of cellular metabolic pathways may alter the T cell lifecycle and can possibly be exploited as therapeutic targets in disease.^16^

Adenosine monophosphate‐activated protein kinase (AMPK) serves as a metabolic master regulator that responds to cellular stimuli of energy depletion (eg, hypoxia or glucose limitation) to induce ATP production via activation of catabolic pathways.^17^ AMPK deficiency in T cells is associated with impaired metabolic adaptation upon nutrient starvation in vitro and impaired T cell function.^18^ However, the mechanism behind AMPK‐mediated regulation of cell metabolism and biology has been a matter of debate.^19^ Importantly, how AMPK links nutrient sensing to T cell activation and effector function has remained elusive, although a number of pharmacological AMPK activators and inhibitors are used both in a clinical setting^20^ and in preclinical disease models.^16^, ^21^, ^22^, ^23^

In this study, we aimed to investigate the mechanism by which AMPK regulates T helper (Th) cell responses in vivo. We show here that AMPK‐deficient T cells exhibit decreased overall expansion irrespective of the induced Th cell subset (Th1, Th2, Th17, or T regulatory cells [Treg]) in vivo. Accordingly, while the quality of the T cell response, that is, Th cell fate decision and clonal diversity, was not changed when compared to wild type (WT) T cells upon in vivo activation, the magnitude of the immune response was equally diminished for all Th cell subsets. These data indicate that loss of AMPK does not impact the program of a particular T helper cell subtype, but influences T cell expansion in general. Interestingly, the effects on CD4 + T cells imposed by AMPK deletion were not due to enhanced apoptosis, impaired migration into effector organs, or T cell exhaustion. Instead, in vivo activated AMPK‐deficient T cells displayed reduced metabolic activity, and impaired expression of ribosomal proteins and ribosomal genes engaged in mRNA translation. Together, these data strongly delineate a novel role of AMPK in coordinating the translational program of activated T cells in vivo depending on the metabolic status of the cell.

## 2 MATERIALS AND METHODS

### 2.1 Mice

Prkaa1f/f mice were obtained from The Jackson Laboratory (MGI: 4836199; Bar Harbor, ME, USA). CD4‐Cre mice were kindly provided by Prof. Wilfried Ellmeier and were originally obtained from Dr Chris Wilson (MGI: 2386448). Prkaa1f/f mice were crossed with CD4‐Cre mice to generate Prkaa1f/f (WT) and Prkaa1f/f CD4‐Cre+ (KO) mice. Rag2–/– mice were kindly provided by Prof. Wilfried Ellmeier and were originally obtained from Taconic (MGI: 1858556, Rensselaer, NY, USA). C57BL/6J mice were obtained from in‐house breeding (originally obtained from The Jackson Laboratory). All mice were used at 6‐8 weeks of age unless otherwise specified. In all in vitro studies and in vivo experiments, WT and KO littermate controls were used for comparison. Animal experimentation protocols were evaluated by the Animal Ethics Committee of the Medical University of Vienna and approved by the Austrian Ministry of Economy and Science (BMWF‐66.009/0027‐II/3b/2014 and BMBWF‐66.009/0308‐V/3B/2018). Animal husbandry and experimentation were performed according to the Federation of Laboratory Animal Science Association guidelines.

### 2.2 Immunoblotting

Immunoblotting was performed as described previously.^24^ Briefly, CD4 + T cells (or CD19 + B cells for the indicated immunoblots) were isolated from splenocytes using magnetic or flow‐cytometric cell sorting and lysed in 2 × Lämmli Buffer (Novex Tris‐Glycine SDS Sample Buffer 2×; Thermo Fisher Scientific Waltham, MA, USA) supplemented with 100 mM DTT (Sigma Aldrich, St. Louis, MO, USA), protease inhibitors (cOmplete ULTRA Tablets; Roche, Basel, Switzerland), and phosphatase inhibitors (PhosSTOP; Roche) for 5 minutes at 99°C. Primary antibodies to phospho‐AMPKα1 (p‐AMPKα1, Thr172), total AMPKα1, phospho‐acetyl‐CoA carboxylase (p‐ACC, Ser79), total ACC, phospho‐TSC2 (p‐TSC2, Thr1462, and Ser1387), total TSC2, phospho‐Raptor (p‐Raptor, Ser792), total Raptor, GAPDH, and pan‐Actin were obtained from Cell Signaling Technology (Cell Signaling Technology, Cambridge, UK). All primary antibodies were used at a dilution of 1:1000. HRP‐conjugated goat anti‐rabbit IgG (Dako, Agilent Technologies, Santa Clara, CA, USA) was used as the secondary antibody at a dilution of 1:2000. Immunoblots were developed using a HRP‐specific substrate (Pierce ECL Western Blotting Substrate; Thermo Fisher Scientific). HRP chemiluminescence signals were detected using a Fujifilm LAS‐4000 image analyzer (GE Healthcare, Chicago, IL, USA) and analyzed using Fiji (ImageJ, https://imagej.net/Fiji).

### 2.3 T cell culture and T helper cell differentiation

For bulk CD4 + T cell culture, WT or KO CD4 + T cells were isolated from splenocytes using CD4 + microbeads (Miltenyi Biotec, Bergisch Gladbach, Germany) and plated on anti‐CD3ε (1 µg/mL; BD Biosciences, Franklin Lakes, NJ, USA) and anti‐CD28 (3 µg/mL; BD Biosciences) precoated Nunc‐treated 48‐ or 96‐well‐plates (Thermo Fisher Scientific) in T cell medium (RPMI 1640 with 11.1 mM glucose [Lonza, Basel, Switzerland] supplemented with 10% fetal calf serum [FCS] [PAA Laboratories, Pasching, Austria], 2 mM l‐glutamine, 100 U/mL penicillin, 0.1 mg/mL streptomycin [Sigma Aldrich], 0.1 mM 2‐mercaptoethanol [Thermo Fisher Scientific], and 25 mM HEPES solution [Sigma Aldrich]). Where indicated, cells were washed with PBS after 2 days and re‐cultured in glucose‐free (RPMI 1640 without glucose [Lonza, Basel, Switzerland] supplemented with 10% dialyzed FCS [Thermo Fisher Scientific], 2 mM l‐glutamine, 100 U/mL penicillin, 0.1 mg/mL streptomycin, 0.1 mM 2‐mercaptoethanol, and 25 mM HEPES) or glutamine‐free medium (RPMI 1640 with 11.1 mM glucose without l‐glutamine [Lonza, Basel, Switzerland] supplemented with 10% dialyzed FCS, 100 U/mL penicillin, 0.1 mg/mL streptomycin, 0.1 mM 2‐mercaptoethanol, and 25 mM HEPES) for 5 hours. Where indicated, CD4 + cells were activated in the presence of rapamycin (10 nM, Cell Signaling Technologies).

For naive T helper cell differentiation, naive CD4 + T cells (CD4 + CD25− CD44 − CD62L+) were isolated from splenocytes via fluorescence‐activated cell sorting using a Sony SH800 cell sorter (Sony Biotechnology, San Jose, CA, USA) after negative magnetic enrichment (EasySep Mouse CD4 + T Cell Isolation Kit; StemCell Technologies, Vancouver, CA). Naive T cells were plated on anti‐CD3ε (1 µg/mL) and anti‐CD28 (3 µg/mL) precoated Nunc‐treated 96‐well‐plates. For Th1 differentiation, naive T cells were stimulated in T cell medium (RPMI 1640 with 11.1 mM glucose and 2 mM l‐glutamine) in the presence of recombinant murine IL‐12 (100 U/mL, Peprotech, Rocky Hill, NJ, USA), recombinant human IL‐2 (100 U/mL, Peprotech), and anti‐IL‐4 (5 µg/mL, clone 11B11; BioLegend, San Diego, CA, USA). To induce Th2 differentiation, naive T cells were stimulated in the presence of recombinant murine IL‐4 (30 U/mL, Peprotech), recombinant human IL‐2 (100 U/mL), and anti‐IFN‐γ (10 µg/mL, clone R4‐6A2, BioLegend). For Th1 and Th2 differentiation protocols, cells were split 1:1 after 2 days and transferred to uncoated 48‐well plates. On day 5, Th1 and Th2 differentiated cells were subjected to intracellular cytokine and transcription factor staining after 4 hours of restimulation with PMA/ionomycin (Cell Stimulation Cocktail; eBioscience, San Diego, CA, USA) and monensin (eBioscience). For Th17 differentiation, cells were stimulated with plate‐bound anti‐CD3ε and anti‐CD28 (1 and 3 µg/mL, respectively) in the presence of TGF‐β (1 ng/mL; R&D Systems, Minneapolis, MN, USA), recombinant murine IL‐6 (20 ng/mL; Peprotech), recombinant murine IL‐1α (10 ng/mL; Peprotech), recombinant murine IL‐1β (10 ng/mL; Peprotech), and recombinant murine IL‐23 (10 ng/mL; Peprotech) for 3 days. Intracellular cytokine staining was performed on day 3 after 4 hours of restimulation with PMA/ionomycin and monensin. To induce Treg differentiation, naive CD4 + T cells were stimulated with plate‐bound anti‐CD3ε and anti‐CD28 (1 and 3 µg/mL, respectively) in the presence of TGF‐β (1 ng/mL) for 3 days. Intracellular transcription factor staining was performed on day 3, as described below.

### 2.4 Treg suppression assay

Treg suppression assays were performed with FACS‐purified WT and KO Treg (CD4 + CD25+) and naive Ly5.1 + Tresponder cells (CD4 + CD25‐CD44‐CD62L+) labeled with V450 proliferation dye (BD Biosciences). T cell depleted splenocytes of C57BL/6 mice served as APCs. Cells were cultured at the indicated ratios in U‐bottom Nunc‐treated 96‐well plates (Thermo Fisher Scientific) in T cell medium and activated with anti‐CD3ε (1 ug/mL, BD Biosciences). Proliferation of the Ly5.1 + Tresponder cell population was assessed after 3 days and is depicted as percent proliferating cells.

### 2.5 Flow cytometric analyses

All staining reactions were performed at 4°C following incubation with FcBlock (anti‐CD16/CD32; eBioscience). For analysis of surface markers, cells were stained in PBS containing 1% BSA (Sigma Aldrich). The following antibodies were used for surface staining: anti‐CD3 (clone 17A2; BioLegend), anti‐CD4 (clone GK1.5; BioLegend), anti‐CD8α (clone 53‐6.7; BioLegend), anti‐CD11b (clone M1/70; BD Biosciences), anti‐CD11c (clone N418; BioLegend), anti‐CD25 (clone PC61; BioLegend), anti‐CD44 (clone IM7; BioLegend), anti‐CD45 (clone 30‐F11; BioLegend), anti‐CD62L (clone MEL‐14; BioLegend), anti‐CD170 (clone 1RNM44N; Thermo Fisher Scientific), anti‐CCR7 (clone 4B12; BioLegend), and anti‐PD‐1 (clone 29F.1A12; BioLegend). For intracellular transcription factor staining, cells were fixed and permeabilized using the Foxp3/Transcription Factor Staining Buffer Set (eBioscience) according to the manufacturer's instructions and intracellularly stained with anti‐Foxp3 (clone FJK‐16S; eBioscience), anti‐T‐bet (clone 4B10; BioLegend), anti‐GATA‐3 (clone L50‐823; BD Biosciences), and anti‐Ki67 (clone 16A8; Biolegend). For intracellular cytokine staining, cells were re‐stimulated for 4 hours with PMA/ionomycin in the presence of monensin before fixation and permeabilization using BD Cytofix/Cytoperm according to the manufacturer's instructions. Cells were then stained with anti‐IL‐17A (clone TC11‐18H10.1; BioLegend), anti‐IL‐17F (clone 9D3.1C8; BioLegend), anti‐IL‐4 (clone 11B11; eBioscience), anti‐IL‐13 (clone eBio13A; eBioscience), and anti‐IFN‐γ (clone XMG1.2; BioLegend). For mitochondrial membrane potential staining, cells were incubated with MitoTracker Orange CMTMRos (Thermo Fisher Scientific) for 20 minutes at 37°C according to the manufacturer's protocol. For the assessment of viability, cells were stained with a fixable viability dye prior to surface staining of antigens (Fixable Viability Dye eFluor780; eBioscience, or Zombie Aqua Fixable Viability Kit; BioLegend). Alternatively, cells were stained for Annexin‐V and 7‐AAD after surface staining according to the manufacturer's protocol (FITC Annexin V Apoptosis Detection Kit with 7‐AAD; BioLegend). Flow cytometric data were acquired on a LSR Fortessa or LSR II (BD Biosciences) and analyzed using FlowJo software (Tree Star, Version 10).

### 2.6 Metabolic assays

#### 2.6.1 Seahorse analysis

WT or KO CD4 + T cells were seeded into 48‐well‐plates precoated with 1 µg/mL anti‐CD3ε and 3 µg/mL anti‐CD28 in T cell medium and stimulated for 48 hours. Subsequently, cells were harvested and plated in Cell‐Tak (Corning Inc, Corning, NY, USA)‐coated Seahorse XF24 microplates (Agilent Technologies, Santa Clara, CA, USA) via centrifugation and cultured in XF Assay media (Seahorse XF assay medium modified DMEM containing 25 mM glucose and 1 mM sodium pyruvate; Agilent Technologies). OCR and ECAR were measured using a Seahorse XF24 Extracellular Flux Analyzer (Agilent Technologies) according to the manufacturer's protocol. Mitochondrial bioenergetics were assessed following the sequential addition of oligomycin (1 µM), fluoro‐carbonyl cyanide phenylhydrazone (FCCP, 1.5 µM) and antimycin A (AA, 1 µM), and rotenone (Rot, 1 µM) (all reagents obtained from Sigma Aldrich) according to the manufacturer's protocol. Raw data were extracted and normalized to total protein concentration.

#### 2.6.2 Metabolomic analysis

WT or KO CD4 + T cells were seeded into 48‐well‐plates precoated with 1 µg/mL anti‐CD3ε and 3 µg/mL anti‐CD28 in T cell medium. Cells were stimulated for 3 days, and then, shock‐frozen in liquid nitrogen. Metabolomic analysis was performed by liquid chromatography‐mass spectrometry (LC‐MS) at Metabolon (Durham, NC, USA) as described previously.^24^ Raw data were extracted and normalized to Bradford protein concentration.

### 2.7 Adoptive T cell transfer colitis

For the development of T cell‐mediated colitis,^25^ Rag2−/− mice were injected intraperitoneally (ip) with 0.5 × 10^6^ naive CD4 + CD25 − CD44 − CD62L+ WT or KO T cells. The purity of transferred CD4 + CD25 − CD44 − CD62L+ naive T cells was approximately 99% (data not shown). Body weights were monitored weekly and mice were checked for clinical signs of disease. After 6 weeks, mice were sacrificed for analysis of immune cell infiltration in the small and large intestine and secondary lymphoid organs. After removal of Peyer's patches, mesenteric lymph nodes (mLN), spleen, small intestine, and colon tissues were isolated. The colon, small intestine, lung, and liver were prepared for histological examination as described previously (“Swiss roll,” see ref. 26) fixed in 7.5% formalin and preceded with a tissue processor (Thermo Fisher Scientific). Histological evaluation was performed on 5 μM thick sections stained with hematoxylin and eosin. Four high power field (HPF) images (ie, 400× magnification) and low‐magnification images (ie, 40× magnification) were collected from each colon, liver, and lung tissue. Four representative HPF images were captured from each tissue and processed using the Olympus cellSens software (Olympus, Tokyo, Japan). Isolation of intraepithelial lymphocytes (IEL) and small intestine was performed as described previously.^27^ Briefly, the small intestine was cut into small pieces. IELs were isolated after digestion of the small intestine with RPMI with 3% FCS, 5 mM EDTA (Thermo Fisher Scientific), and 1 mM DTT for 20 minutes at 37°C with gentle shaking. Digested tissue was filtered through a 70 µm cell strainer and cell suspensions were washed with RPMI containing 10% FCS. Percoll (Sigma Aldrich) density centrifugation was performed to remove dead cells. Viable cells were counted using either trypan blue exclusion or a Coulter Counter (Beckman Coulter, Brea, CA, USA). Cells from all compartments (spleen, mLN, and IEL) were re‐stimulated with PMA/ionomycin in the presence of monensin, as described above, and subjected to flow cytometric analysis. Where indicated, transferred WT naive CD4 + T cells were analyzed at earlier timepoints to assess AMPKα1 activation dynamics in vivo.

### 2.8 Induction of allergic airway inflammation in vivo

House dust mite extract (HDM; extracted from *Dermatophagoides pteronyssinus*) was purchased from Greer Laboratories (Cambridge, MA, USA). For sensitization, WT or KO mice were anesthetized upon ip administration of ketamine (100 mg/kg body weight; Pfizer, New York, NY, USA) and xylazine (5 mg/kg; Bayer, Leverkusen, DE). HDM (or PBS in control mice) was intratracheally (i.t.) delivered (HDM: 1 µg HDM in 40 µL PBS; PBS: 40 µL PBS) at day 0. Mice were intranasally (i.n.) challenged with 10 µg HDM (10 µg in 30 µL PBS) or PBS (30 µL) at day 7‐11. Seventy‐two hours after the last challenge, mice were sacrificed to analyze the allergic phenotype. Blood was collected from the inferior vena cava. Serum was obtained after centrifugation at 1200 rpm for 10 minutes to measure total serum and antigen‐specific IgE levels. Total IgE and HDM‐specific IgE were determined using the ELISA MAX Standard Set Mouse IgE Kit (BioLegend). For analysis of immune cell infiltration, the lungs were isolated as previously described.^28^ Briefly, lungs were excised, minced, and digested in serum‐free RPMI containing Liberase CI (0.05 mg/mL; Roche) and DNase I (0.5 mg/mL; Sigma Aldrich) at 37°C for 60 min. Digested tissue was filtered through a 70 µm cell strainer and cell suspensions were washed with RPMI containing 10% FCS. Red blood cell lysis was performed. Viable cells were counted using either trypan blue exclusion or a Coulter Counter. Cell suspensions were subjected to flow cytometric analysis or antigen‐specific re‐stimulation. For intracellular cytokine staining, 1 × 10^6^ cells were re‐stimulated with PMA/ionomycin in the presence of monensin, as described above, and subjected to flow cytometric analysis. For HDM‐specific restimulation, whole lung cell suspensions were cultured in a 96‐well plate with HDM extract (50 μg/mL). Cell culture supernatants were harvested after 72 hours to analyze cytokine production via Luminex technology according to standard protocols (R&D Systems).

### 2.9 RNA isolation

Splenic or mLN T cells were sorted in PBS. After centrifugation, cells were lyzed in 500 uL TRI Reagent (Sigma Aldrich). RNA was isolated according to the manufacturer's protocol and RNA was dissolved in 8‐20 μL RNAse free water and stored at −80°C until further processing.

### 2.10 mRNA sequencing analysis

RNA was used for sequencing library preparation at the Core Facility Genomics, Medical University of Vienna, using the NEBNext SingleCell/Low Input Library Prep Kit according to the manufacturer's protocols (New England Biolabs, Ipswich, MA, USA). Libraries were QC‐checked using a Bioanalyzer 2100 (Agilent) using the High‐Sensitivity DNA Kit for correct insert size and quantified using the Qubit dsDNA HS Assay (Invitrogen, Carlsbad, CA, USA). Pooled libraries were sequenced on a NextSeq500 instrument (Illumina, San Diego, CA, USA) in 1 × 75 bp single‐end sequencing mode. Approximately 30 million reads were generated per sample. Reads in fastq format were aligned to the mouse reference genome version GRCM38 (mm10 murine reference genome, downloaded from Ensembl on 2018‐09‐28) with Gencode mV23 annotations (murine genome annotations downloaded from Gencode on 2019‐10‐14) using STAR aligner^29^ version 2.6.1a in 2‐pass mode. Reads per gene were counted using STAR, and differential gene expression was calculated using DESeq2^30^ version 1.22.2. One mLN sample from WT T naive Rag2–/– recipients did not meet quality control and was excluded from further analysis.

### 2.11 T cell repertoire sequencing analysis

RNA was used for TCR cDNA library preparation using a TCR profiling Kit (MiLaboratory, Moscow, Russia) according to the manufacturer's protocol with minor modifications. Briefly, cDNA synthesis was performed with primers corresponding to the constant region of the mouse TCR beta chain. 5′ RACE technology was used to add universal adapters with unique molecular identifiers (UMI) to the 5′ end of each cDNA molecule. After cDNA synthesis, two rounds of amplification were used to add standard Illumina adapters and sample barcodes. Libraries were pooled and sequenced on an Illumina MiSeq instrument (paired end, 2 × 150 nt). Raw sequencing reads were processed with MIGEC software^31^ for UMI‐based clustering and error correction. Alignment of V, D, and J segments was performed using MIXCR with default parameters.^32^ Before performing the actual analysis, the MIXCR output was downsampled to the same amount of UMIs in each library using VDJtools.^33^

### 2.12 Data reporting and statistical analysis

No statistical methods were used to predetermine sample size. The experiments were not randomized and the investigators were not blinded during experiments and outcome assessment. The data shown indicate the mean ± SD or SEM (for biological replicates) as specified in the respective figure legends. P‐values were calculated with paired or unpaired two‐tailed Student's *t* test (comparisons between two groups) or with one‐way ANOVA (comparisons between three or more groups), as indicated in the respective figure legends. A *P*‐value below <.05 was considered statistically significant (*), *P* < .01 (**) or *P* < .001 (***).

### 2.13 Data availability statement

Messenger RNA‐sequencing data and TCR beta sequencing data have been deposited in the GEO database under ID code GSE152157.

## 3 RESULTS

### 3.1 AMPK acts as a nutrient sensor and is activated during in vivo T cell responses

**FIGURE 1:**
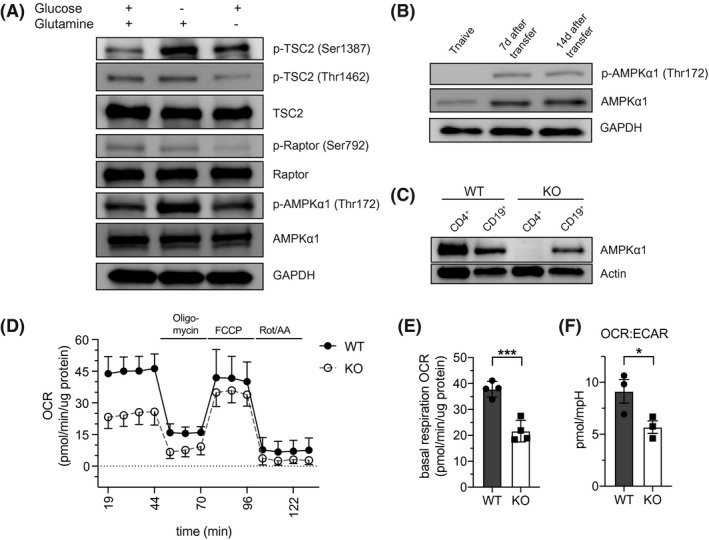
AMPK supports T cell metabolic adaptation and is activated during primary T cell responses in vivo. A, Immunoblot for AMPKα1 activation and activation of mTORC1 regulatory proteins (TSC2 and Raptor) in activated WT cells under nutrient‐replete conditions or after acute (5 hours) glucose or glutamine starvation. One of two independent experiments is shown. B, Immunoblot for AMPKα1 activation (phosphorylation at Thr172) dynamics in vivo. Freshly isolated WT CD4 + T naive cells (before transfer) and CD4 + T cells recovered from the spleen of Rag2–/– recipient mice at the indicated timepoints after adoptive transfer were analyzed. One experiment is shown. C, Immunoblot for AMPKα1 protein expression in splenic CD4 + T cells and CD19 + B cells of WT and KO mice. One of two independent experiments is shown. D, WT or KO CD4 + T cells were activated for 48 hours and oxygen consumption rate (OCR) was determined by Seahorse analysis at baseline and in response to metabolic stimuli. One of three representative experiments performed in quadruplicates is shown. E, Summary of basal OCR of WT and KO CD4 + T cells from one representative experiment as determined in (D). F, OCR:extracellular acidification rate (ECAR) ratio from WT and KO T cells analyzed in three independent experiments as determined in (D). Data shown indicate mean ± SEM or SD; *P < .05, ***P < .001 paired or unpaired student's *t* test. AA, antimycin A; FCCP, fluoro‐carbonyl cyanide phenylhydrazone; Rot, rotenone

To investigate the dependency of activated T cells on the availability of nutrients, we activated CD4 + T cells under nutrient‐replete conditions for 48 hours with subsequent acute glucose or glutamine starvation for 5 hours. Glucose‐deprived CD4 + T cells exhibited increased AMPKα1 activation as indicated by higher levels of p‐AMPKα1 (Thr172) (Figure 1A). Activation of AMPK by glucose starvation resulted in a direct phosphorylation of tuberous sclerosis complex 2 (TCS2) at Ser1387, as described previously^34^, ^35^, ^36^ (Figure 1A). To further investigate if AMPKα1 is also activated in T cells during in vivo activation, we transferred naive CD4 + T cells into Rag2–/– recipient mice and assessed AMPKα1 activation levels at sequential timepoints after adoptive transfer. AMPKα1 activity was not detected in naive CD4 + T cells before transfer, however, we observed increased levels of total AMPKα1 and p‐AMPKα1 (Thr172) in in vivo activated, expanding CD4 + T cells after adoptive transfer (Figure 1B). In sum, these results suggest that both AMPKα1 activity and AMPKα1 expression increase upon T cell activation. To further analyze how AMPK signaling contributes to T cell function, we generated mice with a T cell‐specific deletion of the catalytic AMPKα1 subunit encoded by the PRKAA1 gene using CD4‐Cre mice, where the target gene is deleted at the stage of CD4 + CD8+ thymocytes (Figure 1C, Figure [Link], [Link], [Link], [Link], [Link]A).^37^ Exclusive expression of the AMPKα1‐subunit has been observed in human and murine lymphocytes.^38^ In line with previous results,^18^, ^39^ deletion of AMPKα1 in lymphocytes did not influence T cell homeostasis under resting conditions indicated by normal CD4 + and CD8 + T cell numbers in primary and secondary lymphoid organs (Figure S1B,C). Additionally, AMPKα1‐deficient (KO) T cells did not exhibit aberrant expression of classical surface immune receptors or intracellular markers compared to wild type (WT) T cells (Figure S1D).

When assessing the metabolic status of activated WT and AMPKα1‐deficient T cells, we observed that KO T cells exhibited reduced basal oxygen consumption rate (OCR) (Figure 1D,E). Extracellular acidification rate (ECAR), a surrogate readout for glycolysis, was not changed in KO compared to WT T cells (Figure S1E). However, decreased oxidative metabolism translated into a reduced OCR:ECAR ratio in KO T cells (Figure 1F) and, thus, these data suggest an altered metabolic fingerprint in AMPK‐deficient T cells upon activation. In line with this concept, we observed diminished phosphorylation of acetyl‐CoA carboxylase (p‐ACC at Ser79), a major downstream target of AMPK, in KO T cells (Figure S1A), further suggesting possible metabolic constraints in the absence of AMPK. Given that AMPK has previously been shown to participate in multistep regulation of mTORC1,^34^, ^35^ we assessed the phosphorylation status of mTOR regulatory proteins upon AMPK deletion. Unexpectedly, we detected higher activation levels of inhibitory regulatory‐associated protein of mTOR (Raptor, phosphorylation at Ser792) in KO T cells compared to WT T cells at nutrient replete conditions (Figure S1F). Notably, phosphorylation of Raptor at Ser792 in murine nonhematopoietic tissues has been considered to be strictly dependent on AMPK.^35^ Thus, increased phosphorylation of Raptor at Ser792 in the absence of AMPK possibly indicates that other kinases may compensate for AMPK‐mediated regulation of Raptor activity in T cells. In parallel, we observed increased phosphorylation of tuberous sclerosis complex 2 (TSC2, phosphorylation at Ser1387) upon AMPK deletion (Figure S1F). Conversely, however, we also observed higher levels of TSC2 phosphorylation at Thr1462, a phenomenon that may be indicative of increased Akt‐signaling, in KO T cells compared to WT cells. It is important to note that a similar phosphorylation status of mTORC1 regulatory proteins (ie, both increased phosphorylation of TSC2 at Ser1387 and Thr1462) was observed when WT and KO T cells were activated in the presence of the mTOR inhibitor rapamycin (Figure S1F), and, thus, these observations might also illustrate a regulatory feedback mechanism upon AMPK deletion given that multiple upstream inputs feed into mTORC1. Altogether, these results suggest a prominent function of AMPK in coordinating pro‐growth and anti‐growth cellular stimuli although the precise role of AMPK‐deficiency on the delicate and multistep regulation of mTORC1 remains still elusive.

### 3.2 AMPK is not required for T cell fate decision or Treg suppressive function

**FIGURE 2:**
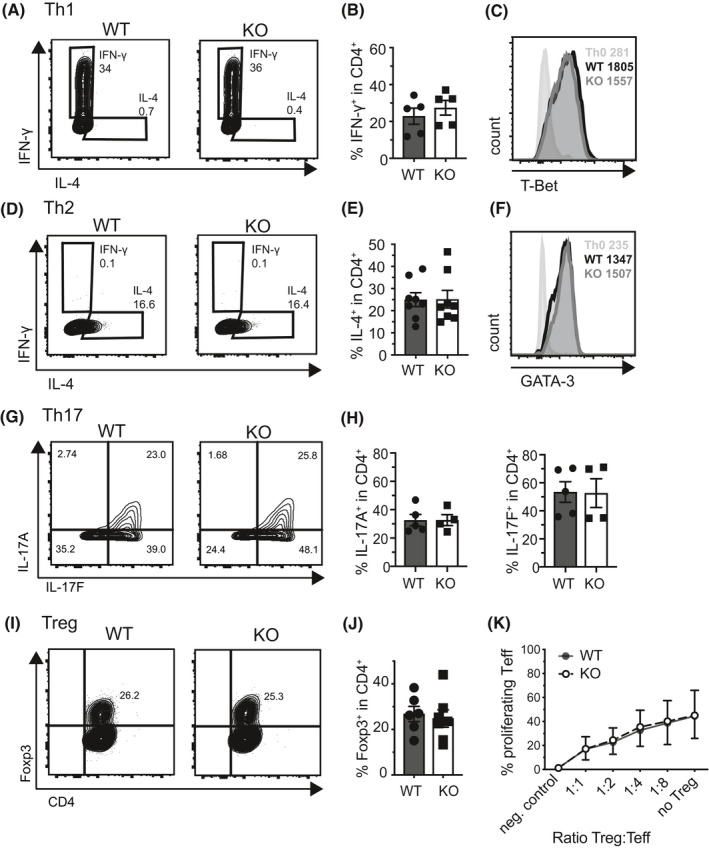
AMPK does not regulate the fate decision of naive CD4 + T cells. A, IFN‐γ production of Th1 polarized naive CD4 + T cells of WT and KO mice after 5 d of cell culture. Panel (B) indicates the summary of (A) of three independent experiments with n = 1‐2 mice per genotype per experiment. C, Representative histogram of intracellular transcription factor staining of Th1 polarized cells at d5 of cell culture. D, IL‐4 production of Th2 polarized naive CD4 + T cells after 5 d of cell culture. Panel (E) shows the summary of (D) of four independent experiment with n = 1‐2 mice per genotype and experiment. Panel (F) shows one representative transcription factor staining of Th2 polarized cells at d5 of cell culture. G, Production of IL‐17A and IL‐17F of Th17 polarized naive CD4 + T cells after 3 d of cell culture. Panel (H) indicates the summary of Panel (G) of three independent experiments with n = 1‐2 mice per genotype per experiment. Panel (I) shows one representative transcription factor staining for Foxp3 in Treg polarized naive CD4 + T cells after 3 d of cell culture. Panel (J) depicts the summary of (I) of five independent experiments with n = 1‐2 mice per genotype per experiment. K, Treg suppression assays of WT and KO Treg with the indicated ratios of Ly5.1 + naive T responder cells (n = 4 mice per genotype in three independent experiments). Percentage proliferating Teff indicate the percent of proliferating cells within the Tresponder population (Ly5.1+). Data shown indicate mean ± SEM. *P < .05, unpaired student's *t* test

Since T cell metabolism frequently follows T cell fate decision and vice versa, we were interested to investigate how loss of AMPK may impact on Th cell differentiation. Interestingly, and in contrast to previous publications using pharmacological targeting of AMPK,^14^ T cell‐specific AMPK deficiency did not translate into impaired Th cell differentiation toward Th1 (Figure 2A‐C), Th2 (Figure 2D‐F), Th17 (Figure 2G‐H), or induced regulatory T cells (iTreg) in vitro (Figure 2I‐J). Additionally, although high levels of AMPK activation are proposed to be a signature of Treg,^4^ AMPK‐deficient Treg were not impaired in their suppressive capacity in vitro (Figure 2K), indicating that Treg function is independent of AMPK.

### 3.3 AMPK is required for proper expansion of Th1, Th17, and Treg cells under inflammatory conditions in vivo

**FIGURE 3:**
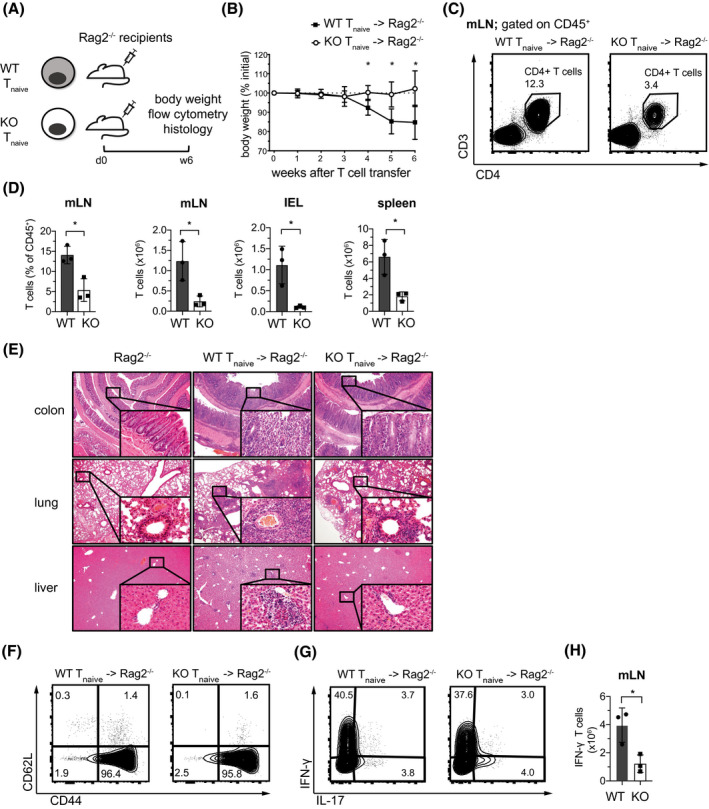
AMPK‐deficient effector T cells show decreased in vivo expansion in a model of T cell transfer colitis. A, Graphical scheme of the experimental setup. B, Body weight (% initial) of Rag2–/– mice receiving either 0.5 × 10^6^ WT or KO naive CD4 + T cells at the indicated time points (n = 3‐4 mice per group representative of three independent experiments). Panel (C) shows a representative contour plot depicting the frequency of CD3 + CD4 + T cells in the mLN at 6 weeks after transfer. Panel (D) shows the relative and/or absolute frequency of CD3 + CD4 + T cells infiltrating the mLN, IEL, or spleen at 6 weeks after transfer (n = 3 mice per group, representative of three independent experiments). In panel (E), organ infiltration was assessed on H&E stained tissue sections of the indicated organs (40× and 400× magnification in the lower right quadrant) in Rag2–/– mice that received PBS, WT, or KO T cells. Representative images of three independent experiments with n = 3‐4 mice per group are shown. Panel (F) shows the expression of CD62L versus CD44 within the CD4 + T cell population at 6 weeks after transfer. Panel (G) shows a representative contour plot depicting the production of IFN‐γ versus IL‐17 at 6 weeks after transfer. Panel (H) shows the quantification of Panel (G) (n = 3 mice per group, representative of three independent experiments). Data shown indicate mean ± SD; *P < .05, unpaired student's *t* test

Given that in vitro T cell stimulation may not necessarily reflect complex in vivo situations in terms of nutrient use and activation dynamics, we employed several in vivo models to investigate the impact of AMPK deficiency on the generation of T cell‐dependent immune responses under inflammatory conditions. First, we used a T cell transfer model of chronic colitis^25^ where gut inflammation is induced by adoptively transferred CD4 + T cells. Rag2–/– recipients received either naive CD4 + T cells of WT or KO mice, and disease progression was monitored using body weight loss as a clinical marker for disease. Mice were sacrificed 6 weeks after transfer, and the presence of inflammatory CD4 + T cells within lymphoid and nonlymphoid compartments was analyzed (Figure 3A). Rag2–/– mice that received WT T cells continuously lost body weight starting from week 4 after T cell transfer. Rag2–/– mice that received KO T cells, in contrast, were able to maintain their body weight (Figure 3B). Moreover, mice that lost more than 20% of their body weight were sacrificed according to standard experimental protocols. While four out of ten Rag2–/– mice that received naive WT donor T cells reached this clinical endpoint, none of the Rag2–/– mice that received naive KO donor T cells had to be sacrificed (Figure [Link], [Link], [Link], [Link], [Link]A). Consistent with the loss in body weight, Rag2–/– recipients of WT T cells developed more severe disease with massive T cell organ infiltration in multiple organs including the spleen, mesenteric lymph nodes (mLN), and the intestinal intraepithelial compartment, while in KO T cell recipients only mild inflammation was observed (Figure 3C‐E). To visualize the quality of the induced immune response, we quantified cytokine production and expression of activation markers of adoptively transferred CD4 + T cells. Infiltrating KO T cells showed similar relative frequencies of activated T effector and T naive cells compared to WT T cells (Figure 3F), indicating that the observed reduction in T cell infiltration in KO T cell recipients was not due to an impaired T effector/memory transition. In accordance with our in vitro data, we observed normal Th1, Th17, and Treg differentiation in vivo (Figure 3G, Figure S2B). However, the absolute numbers of interferon (IFN)‐γ‐producing T cells recovered from the gut were reduced in KO versus WT T cell Rag2–/– recipients (Figure 3H). Similarly, AMPK‐deficient pathogenic Th17 cell and immunosuppressive Treg populations showed a strong trend toward reduction at 6 weeks after transfer (Figure S2C). Because loss of AMPK signaling has been linked to induction of premature senescence and cell death,^18^, ^40^ we assessed the expression of cellular markers of T cell exhaustion and analyzed cell death ex vivo. We found no aberrant expression of exhaustion markers as indicated by normal PD‐1 expression in KO T cells in the spleen and gut, compared to WT T cells (Figure S2D). Staining for Annexin V and 7‐AAD further revealed no evidence of enhanced apoptosis or necrosis in recovered gut infiltrating KO T cells, compared to WT T cells (Figure S2E).

### 3.4 AMPK shapes the quantity of the T effector cell immune response but not the quality of proliferation

Given that AMPK deficiency seemed to impair the absolute magnitude of T cell expansion but not the quality of the induced immune response (Th cell distribution or T effector cell generation), we analyzed whether AMPK was required for T cell expansion upon in vivo stimulation. As failure of clonal expansion would result in a distortion of the T cell receptor (TCR) repertoire, we performed TCR beta chain sequencing of mLN and splenic CD4 + T cells 6 weeks after adoptive transfer into Rag2–/– recipients (Figure [Link], [Link], [Link], [Link], [Link]A). The distribution of TCR beta clonotype sizes was similar among isolated WT and KO T cells after 6 weeks of in vivo proliferation (Figure S3B). In other words, the top clone made up 10.1 ± 3.6% (mean ± SD) of the total repertoire in WT and 7.1 ± 4.2% in KO, and the top 10 clones made up 26 ± 5.8% and 25.9 ± 8% of the repertoire in WT and KO, respectively. Therefore, we hypothesized that the selection of clones is similar in WT and KO T cells, but the quantity of clonal expansion (ie, the absolute magnitude) may be attenuated in AMPK‐deficient T cells. When plotting the clonotype frequencies observed in the two different effector organs of each mouse (ie, mLN vs spleen), we found that clonotypes were similar in spleen and mLN in WT and KO mice. Accordingly, in both genotypes, clones that were frequent in the spleen were also similarly frequent in the mLN compartment, indicating that the impaired functional outcome in vivo is due to an absolute reduction of T cells in the secondary lymphoid organs and the gut, and not due to migration deficits of AMPK‐deficient T cells (Figure S3C). To further corroborate our findings, we examined the presence of the same nucleotide TCR beta sequences in the TCR repertoires of different mice. Clones were shared between the mLN and spleen of each individual mouse and not between different mice, indicating that no a priori skewing of the TCR repertoire was present before T cell transfer in either WT or KO mice (Figure S3D). These results suggest that AMPK regulates the quantity (clone size in terms of absolute numbers) of a T cell‐dependent immune response, but does not impact on clonal diversity.

### 3.5 AMPK orchestrates T cell metabolic and translational adaptation upon activation

**FIGURE 4:**
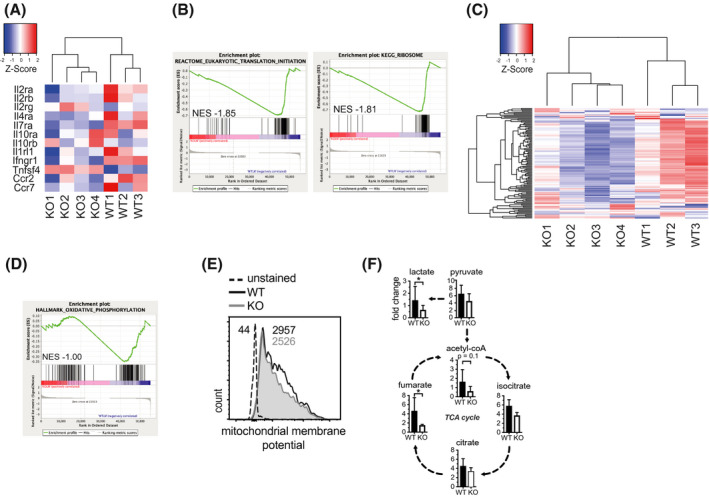
AMPK controls the translational and metabolic fitness of activated CD4 + T cells. A, Heatmap of cytokine receptor expression in WT and KO mLN samples as determined by RNAseq analysis. B, GSEA depicting underrepresented gene sets in KO versus WT T cells 6 weeks after adoptive transfer. C, Heatmap of individual genes of the gene set eukaryotic translation initiation derived from Panel (B). D, Enrichment plot for the gene set oxidative phosphorylation as determined by GSEA. E, Representative histogram of the quantification of mitochondrial membrane potential in WT and KO T cells at 6 weeks after transfer. Numbers indicate the respective MFI values of Mitotracker Orange CMTMRos. F, depicts the relative abundance (fold change) of hallmark metabolites in KO compared to WT activated T cell extracts normalized to Bradford protein concentration. Data shown in Panel (A)‐(E) were obtained in one experiment or are representative of one experiment with n = 3‐4 mice per group. Data shown in Panel (F) indicate mean ± SD of n = 4 independent experiments with n = 5‐10 pooled mice per replicate per group. *P < .05, paired student's *t* test with Welch correction

As AMPK deficiency in T cells resulted in a quantitatively attenuated but homeostatic effector cell expansion in vivo, we aimed to determine which cellular processes limited the proliferative capacity of AMPK‐deficient T cells upon antigenic challenge. Therefore, we analyzed the mRNA expression profiles of sorted mLN and splenic T cells 6 weeks after adoptive T cell transfer into Rag2–/– recipients (Figure [Link], [Link], [Link], [Link], [Link]A). This mechanistic approach allowed us to quantify changes in the cellular transcriptome during *bona fide* T cell activation in vivo. Gene expression profiles in mLN and splenic T cells correlated well between mice and genotypes (Figure S4B‐D). Differentially expressed genes included cytokine and chemokine receptors (including interleukin 2 receptor α [IL2Rα] and IL7Rα) that were markedly reduced in KO T cells (Figure 4A, Figure S4D), although expressed at normal levels in resting unstimulated T cells (Figure S1D). Thus, AMPK‐deficient T cells may be deprived of pro‐growth signals at sites of inflammation during adaptive immune responses.

To further explore the biological significance of AMPK in T cells, we employed gene set enrichment analysis (GSEA) of our mRNA‐seq data to identify enriched or underrepresented functional pathways in our samples. The top hits that were underrepresented in KO T cells included ribosomal gene sets (Figure 4B). This phenotype was evident for gene sets engaged in ribosome biogenesis, including the expression of ribosomal proteins and gene sets engaged in mRNA translation initiation (Figure 4B,C). With translation representing the cell's most energy‐consuming process, we asked whether AMPK‐deficient T cells display reduced metabolic activity, which might explain why KO T cells decrease their translational activity. Using GSEA, we observed that genes engaged in oxidative phosphorylation appeared underrepresented in KO versus WT T cells (Figure 4D), suggesting that KO T cells exhibit both limited metabolic activity and a quantitatively reduced translational apparatus when activated in vivo. In order to further validate these mRNA expression profiles, we quantified mitochondrial membrane potential of in vivo activated T cells at 6 weeks after transfer and observed reduced mitochondrial membrane potential staining indicative of a reduced oxidative metabolism in KO T cells compared to WT T cells (Figure 4E). Subsequently, we performed targeted metabolomic analysis of activated WT and KO T cell extracts using liquid chromatography‐mass spectrometry (LC‐MS). Of the 569 analyzed biochemicals, 80 were significantly less abundant in KO T cells with another 49 trending toward significance (ie, a p‐value between 0.05 and 0.10). Importantly, we observed a reduction in tricarboxylic acid cycle (TCA) cycle intermediates in activated KO T cells compared to WT T cells as well as decreased levels of intermediates of catabolic amino acid and fatty acid metabolism (Figure 4F, Figure S4E‐F). Collectively, these data reveal an essential role for an AMPK‐dependent program in T cells to fuel the metabolic and translational demands of antigen‐challenged T cells in vivo.

### 3.6 AMPK is required for the induction of Th2 dependent disease in vivo

**FIGURE 5:**
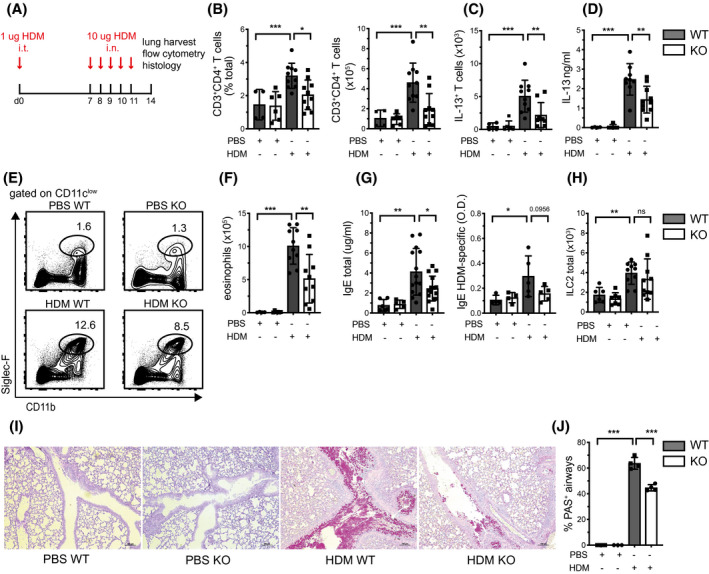
Loss of AMPK in T cells protects against allergic airway inflammation. A, shows the protocol for the induction of HDM‐induced allergic airway inflammation. Panel (B) shows the relative and absolute frequency of lung infiltrating CD3 + CD4 + T cells (pooled data from two independent experiments with n = 2‐6 mice per group). C, shows the frequency of lung Th2 cells at 14 d. D, depicts IL‐13 production of lung suspensions after antigen‐specific restimulation with HDM‐extract in vitro for 3 d. E, shows one representative staining of lung suspension for eosinophils at 14 d. F, indicates the absolute numbers of eosinophils in the lung at 14 d. Panel (G) depicts serum total IgE levels (left panel) and HDM‐specific IgE levels (right panel) determined by ELISA at 14 d. H, shows the frequency of ILC type 2 cells in the lung at 14 d. Panel (I) shows representative PAS stainings of lung sections. J, indicates the percentage of PAS^+^ airways in slides derived from (I). Data shown indicate mean ± SD and are pooled from two independent experiments with n = 3‐6 mice per group, except for panel (G) where HDM‐specific IgE production represents data from one experiment with n = 3‐5 mice per group. Data shown indicate mean ± SD or mean ± SEM for panel (G) and (J). *P < .05, **P < .01, ***P < .001, one‐way ANOVA

After establishing a marked reduction of organ‐infiltrating T cells in a model of Th1/Th17/Treg activation, we were interested in systematically probing whether this obvious impairment was a global phenomenon affecting all Th cell subtypes. Thus, we evaluated whether loss of AMPK in T cells also impairs the function of type 2 T helper cells in vivo using a murine model of house‐dust mite (HDM)‐induced allergic airway inflammation. WT and KO mice were intratracheally (i.t.) sensitized and intranasally (i.n.) challenged with HDM antigen,^28^ and infiltration of the lungs with pathogenic T cells and eosinophils was assessed after 14 days (Figure 5A). HDM‐challenged KO mice exhibited less overall T cell and pathogenic Th2 cell infiltration into the lung compared to WT mice (Figure 5B,C), mirroring previously observed effects in the colitis model. This effect was again independent of T cell exhaustion or cell death (Figure [Link], [Link], [Link], [Link], [Link]A‐D). Similarly, we found reduced production of interleukin (IL)‐13 from HDM‐re‐stimulated lung cells from KO mice compared to WT mice (Figure 5D). The impaired Th2 phenotype was accompanied by reduced lung eosinophilia (Figure 5E,F) and reduced levels of total serum IgE and, although not meeting statistical significance, HDM‐specific IgE (Figure 5G). These data indicate attenuated systemic Th2‐driven allergic inflammation in the absence of AMPK in T cells. Since type 2 innate lymphoid cells (ILC2) play a pivotal role at mucosal barriers and may quickly and forcefully respond to allergens, we wanted to exclude that our observed phenotype was due to a dysregulated ILC2 compartment in KO mice. We found comparable numbers of ILC2 cells at baseline and after allergen challenge in WT and KO mice, indicating that the reduced allergic phenotype was T cell‐dependent (Figure 5H). In line with the reduced lung Th2 responses observed, periodic acid‐Schiff (PAS) staining of lung sections revealed lower numbers of mucus‐positive airway epithelial cells in HDM‐exposed KO mice compared to WT mice (Figure 5I,J). Interestingly, we found a reduced number of lung Treg cells upon allergen challenge in AMPK‐deficient mice (Figure S5E), which is in line with our previous observations in the colitis model. However, attenuated Treg accumulation in effector organs of KO mice might also be related to a reduced need for suppression in response to reduced organ damage. Collectively, these results illustrate a T cell‐intrinsic need for proper AMPK activity for the quantitative dynamics of primary T cell responses in vivo. In our hands, different Th cell subsets were similarly sensitive to loss of AMPK, with reduced accumulation in peripheral organs and reduced severity of disease.

## 4 DISCUSSION

Herein, we demonstrate that the metabolic sensor AMPK regulates the magnitude of T cell‐dependent immune responses while being dispensable for the quality of primary T cell responses in vitro and in vivo. Consistent with the notion that cellular metabolic adaptation is required to fuel an adequate T cell response, defective oxidative metabolism in AMPK‐deficient T cells translated into a quantitative reduction of organ‐infiltrating T cells in our in vivo models. From a functional point of view, we argue that AMPK serves as a checkpoint that integrates information on T cell metabolism and nutrient supply to increase T cell translational activity that ultimately fuels T cell expansion in vivo.

Translation and protein synthesis represent highly energy‐consuming processes, and, therefore, seem tightly linked to the metabolic status of the cell. Notably, the relevance of proper expression and function of the translational apparatus in immunity and cancer has gained increased attention.^41^, ^42^ Increases in transcription and translation of mRNAs that encode components of the translation machinery, including ribosomal proteins, have been described recently during the expansion phase of an antigen‐specific CD8 + T cell response in vivo in response to lymphocytic choriomeningitis virus (LCMV) infection.^43^ For CD8 + T cells, it has been observed that both the expression of IL7Rα and IL2Rα positively correlate with the translational activity of activated CD8 + cells, indicating a prominent role of these cytokines in maintaining translational fitness during T cell expansion in vivo. In line with these observations, the strength of cytokine signaling received by activated T cells has long been known to shape the magnitude of clonal expansion.^44^ Accordingly, we observed decreased expression of pro‐growth cytokine receptors in activated KO T cells compared to WT T cells upon antigenic challenge in vivo. The limited potential to receive pro‐growth cytokine signals in AMPK‐deficient T cells might, therefore, contribute to our observed phenotype. When considering AMPK‐mediated regulation of immune cell receptor expression, conflicting evidence has been reported in the literature. As such, AMPK was shown to inhibit chemokine receptor expression in macrophages via an nuclear factor kappa‐light‐chain‐enhancer of activated B cells (NF‐κB)‐mediated pathway.^45^ Similarly, one study has observed that co‐culture of T cells with mesenchymal stromal cells suppresses CD25 mRNA translation in T cells, a phenomenon that has been associated with increased AMPK activation in co‐cultured T cells.^46^ In contrast, other evidence suggests that pharmacologic inhibition of AMPK in T cells strongly suppresses CD25 expression in a dose‐dependent manner.^47^ These so far controversial observations emphasize a need for a more precise investigation of this subject in the future. In sum, we can only speculate on whether the reduced cytokine receptor expression is a primary effect of AMPK deficiency in T cells or if it is secondary due to the observed globally decreased translational activity.

In a recent study, Ma et al investigated the metabolic status of in vivo activated CD8 + T cells using ^13^C‐based stable isotope tracing, revealing an upregulation of glycolytic and TCA cycle oxidative metabolism upon *Listeria monocytogenes* infection in vivo, compared to naive CD8 + T cells. By combining ex vivo metabolomic and proteomic profiling, the authors linked the metabolic program of activated T cells to pathways associated with cellular proliferation, including enhanced cell cycle progression, induction of ribosome biogenesis, and initiation of mRNA translation.^48^ Conversely, downregulation of components of the translational apparatus and a defective TCA cycle have been observed during the process of CD8 + T cell exhaustion in vivo.^49^ Thus, our data support the hypothesis that metabolic adaptation, and an active and uncompromised translational machinery represent key prerequisites for robust and functional antigen‐specific T cell responses in vivo. Notably, studies investigating these processes in CD4 + T cells have been lacking and, therefore, this study provides the first evidence that CD4 + T cell metabolic activity and translational fitness during in vivo expansion might be coordinated in similar patterns. However, how T cells adopt AMPK signaling in order to connect T cell metabolism to T cell translational fitness upon activation remains largely unknown. Further studies are needed to investigate in detail whether the translational fitness of T cells follows the expression of cytokine receptors or vice versa, and if CD4 + antigen‐specific T cell responses follow the same translation kinetics as CD8 + T cells.

Our data suggest that both levels of total AMPKα1 and p‐AMPKα1 increase in WT T cells when activated in vivo and we can, therefore, only speculate on whether changes in AMPKα1 expression or enhanced AMPKα1 activity per se drive the metabolic and translational adaptations in expanding T helper cells. However, dynamic increases in total protein expression and in the activity of nutrient‐sensitive and pro‐growth signaling pathways, such as mTORC1 or MYC, have been described in a recent study that applied integrative proteomics and phosphoproteomics profiling of in vitro activated T cells.^50^ Thus, AMPKα1 expression and activation may be part of a similarly regulated molecular machinery that contributes to nutrient‐sensing and to the regulation of cell growth upon antigen receptor ligation.

Our results further confirm existing evidence suggesting that dysregulation of upstream central metabolic rheostats that link metabolic cues to translation preferentially impair T cell differentiation and effector function on a general level. As for the kinase mTOR, several lines of evidence highlight that a delicate control of mTOR signaling is essential for lineage fate decision and Th cell function,^51^, ^52^, ^53^ and that tightly controlled mTOR signaling networks regulate both T effector cell and Treg cell differentiation and function.^54^ Liver kinase B1 (LKB1), the major upstream kinase of AMPK, is a metabolic switch that directs T effector cell and Treg cell metabolism. Several studies have demonstrated that the enzyme is critical for the survival, proliferation, and lineage integrity of CD4 + T cells. However, these results appeared to be largely independent of AMPK and depended on the respective knockout model used.^39^, ^55^, ^56^ Importantly, AMPK can also be activated by signaling via calcium/calmodulin‐dependent protein kinase β (CaMKKβ), which switches on AMPK in response to TCR‐mediated calcium flux.^38^ Additionally, AMPK may be activated via transforming growth factor‐β (TGF‐β)‐activated kinase‐1 (TAK1) upon cytokine signaling.^57^, ^58^ Thus, AMPK might represent a nutrient‐sensing checkpoint that acts to promote and provide support for Th cell expansion in general, and may act at least partly distinct from LKB1 and mTORC1.

Our results are also in line with findings from another recent study that observed a quantitatively impaired antitumor response of AMPK‐deficient CD8 + T cells in vivo. Mechanistically, the authors linked an aberrant activation of serine/threonine protein phosphatases, such as protein phosphatase 2A, to increased rates of apoptosis in AMPK KO CD8 + T cells within the tumor microenvironment.^59^ Increased cell death of AMPK‐deficient T cells has also been observed in response to nutrient deprivation ^18^ or in response to metabolic inhibitors in vitro.^60^ Although we and others^18^ did not detect an induction of apoptosis in AMPK‐deficient T cells in vivo, and our mRNA‐seq data argue against a role of aberrant induction of apoptosis in the absence of AMPK, we cannot definitively rule out that loss of AMPK primes T cells for activation‐induced cell death at sites of inflammation. However, the fact that we detected a similar reduction of T cells in the spleen and effector organs (eg, the gut) during in vivo activation also argues against a site‐specific induction of apoptosis at sites of inflammation.

Finally, normal Th cell fate decision in AMPK‐deficient T cells in vitro and in vivo might seem in contrast to previous studies that have used pharmacological AMPK activators and/or inhibitors for modulation of Th cell fate decision.^4^, ^14^, ^22^ In light of recent studies assessing off‐target effects of AMPK‐activators,^47^, ^61^ further investigations are needed to distinguish between AMPK‐dependent and AMPK‐independent effects of those compounds that may affect Th lineage decision and function.

In summary, our results suggest that the cellular energy balance is tied to the T cell translational apparatus via an AMPK‐mediated checkpoint that ultimately orchestrates the quantitative dynamics of primary T cell responses.

## CONFLICT OF INTEREST

The authors declare that they have no conflict of interest.

## AUTHOR CONTRIBUTIONS

K.A. Mayer, G.A. Gualdoni, and G.J. Zlabinger designed the study. K.A. Mayer, G.A. Gualdoni, G.J. Zlabinger, U. Smole, and N. Boucheron designed experiments. K.A. Mayer performed the in vitro and in vivo experiments and analyzed the data with help from U. Smole, C. Zhu, A. Christamentl, and P. Waidhofer‐Söllner, S. Derdak and M. Bilban performed global mRNA sequencing and analyzed the data. A.A. Minervina, M. Salnikova, and I.Z. Mamedov performed TCR beta sequencing and analyzed the data. N. Witzeneder performed Seahorse analysis and analyzed the data. N. Boucheron, G. Hoermann, K.G. Schmetterer, W. Ellmeier, W.F. Pickl, and M. Trauner contributed mice, critical reagents and/or provided important scientific input. K.A. Mayer, G.A. Gualdoni, and G.J. Zlabinger wrote the manuscript. All authors carefully read and revised the manuscript and agreed to its final version.

## Supporting information

Fig S1

Fig S2

Fig S3

Fig S4

Fig S5
