# Supplementary material for: The energy sensor AMPK orchestrates metabolic and translational adaptation in expanding T helper cells
Source: FASEB J. 2021 Mar 14;35(4):e21217. doi: 10.1096/fj.202001763RR (PMC8252394; doi:10.1096/fj.202001763RR)
Supplement: Supplementary file 1 — Fig S1 [file FSB2-35-0-s002.docx]

# Supplemental Figure 1


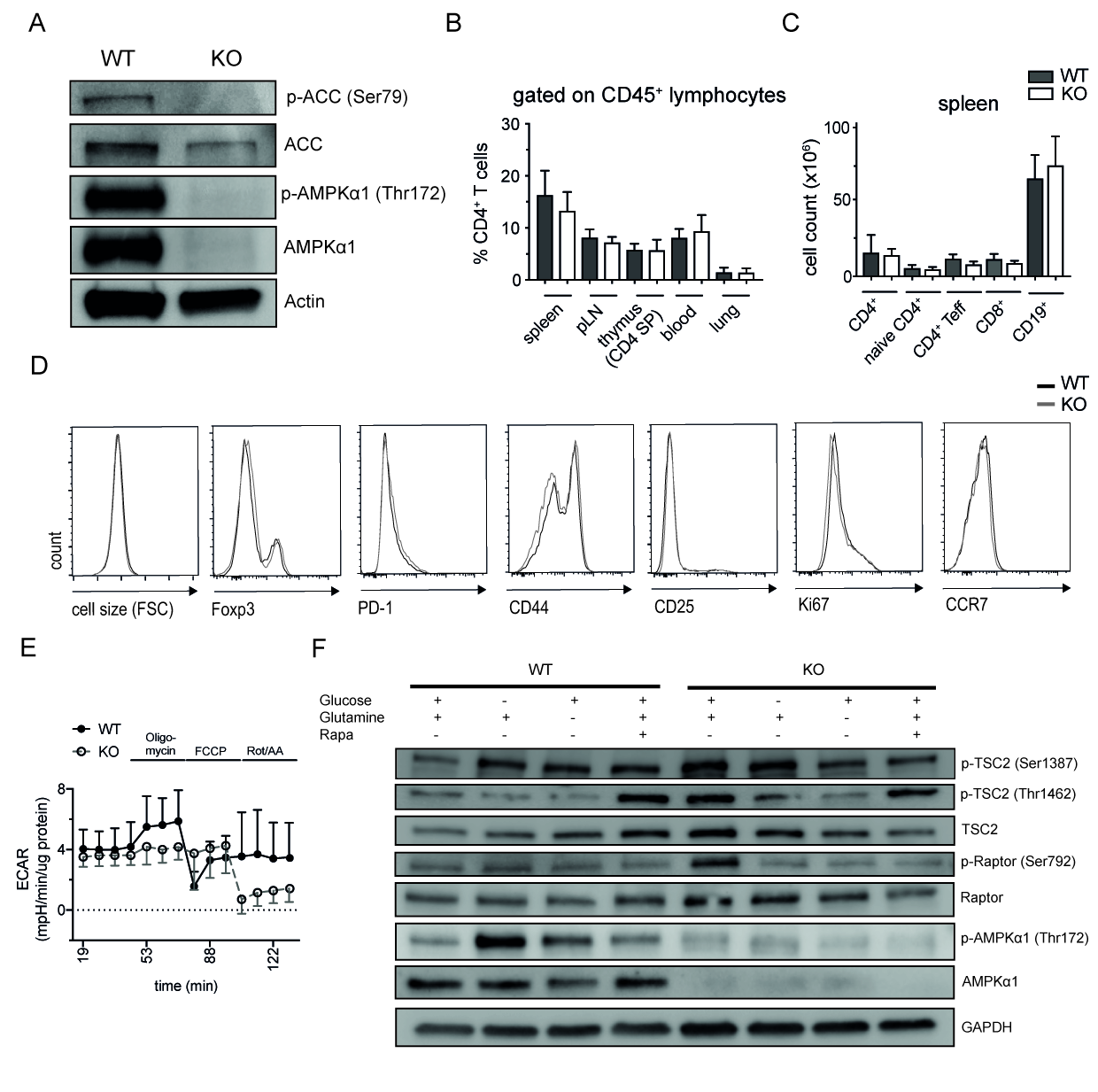


**Supplemental Figure 1. AMPK does not regulate T cell homeostasis but may impact on T cell metabolism and mTORC1**

A) Immunoblot for AMPKα1 activation (Thr172) and phosphorylation of AMPK downstream targets in activated WT and KO T cells. One of two independent experiments is shown. Relative (B) and absolute (C) frequencies of CD4+ and/or CD8+ T cells in the indicated organs in naive WT and KO mice. Data shown indicate mean ± SD of 4 independent experiments with n=2-3 mice per group. D) Histograms showing the expression of the indicated markers in splenic CD4+ T cells of WT and KO mice. Data shown are representative of 3 independent experiment with n= 2-3 mice per group. E) Extracellular acidification rate (ECAR) of activated WT and KO cells as determined by Seahorse Analysis (basal and in response to metabolic stimuli). F) Immunoblot for AMPKα1 activation and activation of mTORC1 regulatory proteins (TSC2 and Raptor) in activated WT or KO cells under nutrient-replete conditions, after acute glucose or glutamine starvation or activated in the presence of the mTOR inhibitor rapamycin (10nM). AA, antimycin A; FCCP, fluoro-carbonyl cyanide phenylhydrazone; Rot, rotenone.
