# Supplementary material for: The energy sensor AMPK orchestrates metabolic and translational adaptation in expanding T helper cells
Source: FASEB J. 2021 Mar 14;35(4):e21217. doi: 10.1096/fj.202001763RR (PMC8252394; doi:10.1096/fj.202001763RR)
Supplement: Supplementary file 2 — Fig S2 [file FSB2-35-0-s003.docx]

# Supplemental Figure 2

**
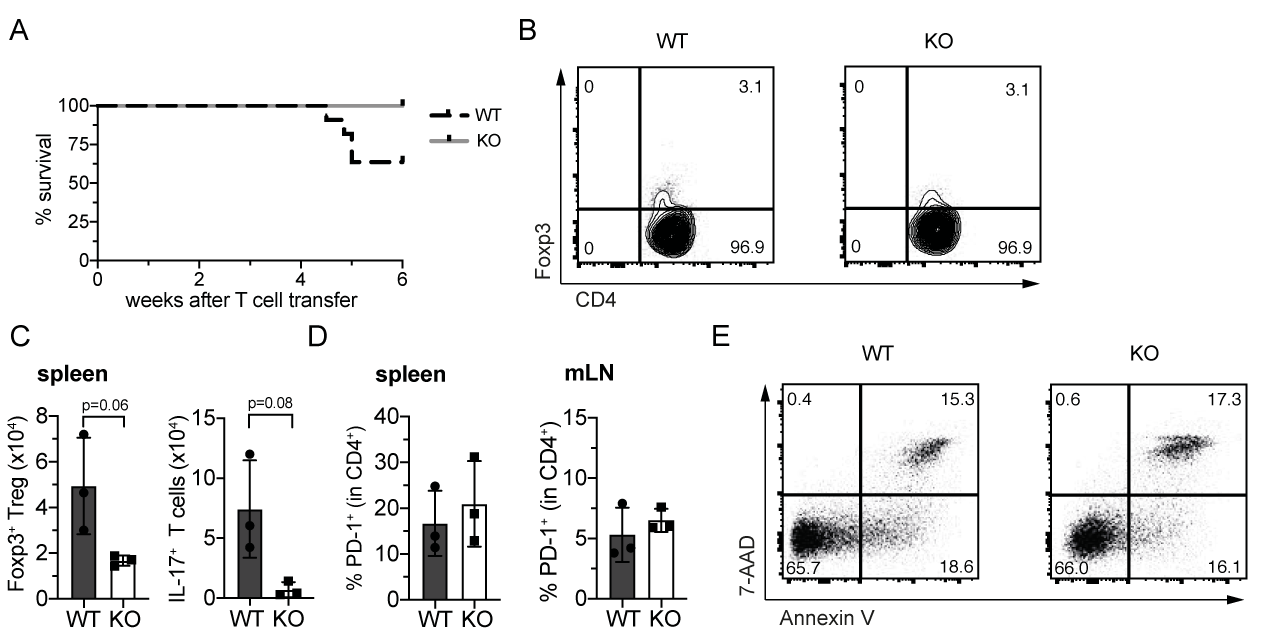
**

**Supplemental Figure 2. Loss of AMPK alleviates T-cell mediated organ infiltration after adoptive transfer *in vivo.***

A) Survival of Rag2–/– mice receiving either 0.5 x 10^6^ naive WT or KO T cells. B) Representative Foxp3 staining gated on CD45+CD3+CD4+ T cells in the mLN at 6 weeks after transfer. C) Absolute counts of splenic Foxp3+ Treg or IL17+ Th17 cells at 6 weeks after transfer. D) Percentage of PD-1+ CD4+ T cells in the indicated organs at 6 weeks after transfer. E) Representative Annexin-V vs. 7-AAD staining of mLN CD4+ T cells 6 weeks after adoptive transfer. Data shown in A) are pooled data from 3 independent experiments with n=3-4 mice per group. Data shown in B) and E) are representative of two independent experiments with n=3-4 mice per group Data shown in C) and D) indicate mean ± SD of n=3 mice and are representative of two independent experiments with n=3-4 mice per group. * *P*<0.05, unpaired student’s t test.
