# Supplementary material for: The energy sensor AMPK orchestrates metabolic and translational adaptation in expanding T helper cells
Source: FASEB J. 2021 Mar 14;35(4):e21217. doi: 10.1096/fj.202001763RR (PMC8252394; doi:10.1096/fj.202001763RR)
Supplement: Supplementary file 3 — Fig S3 [file FSB2-35-0-s005.docx]

# Supplemental Figure 3

**
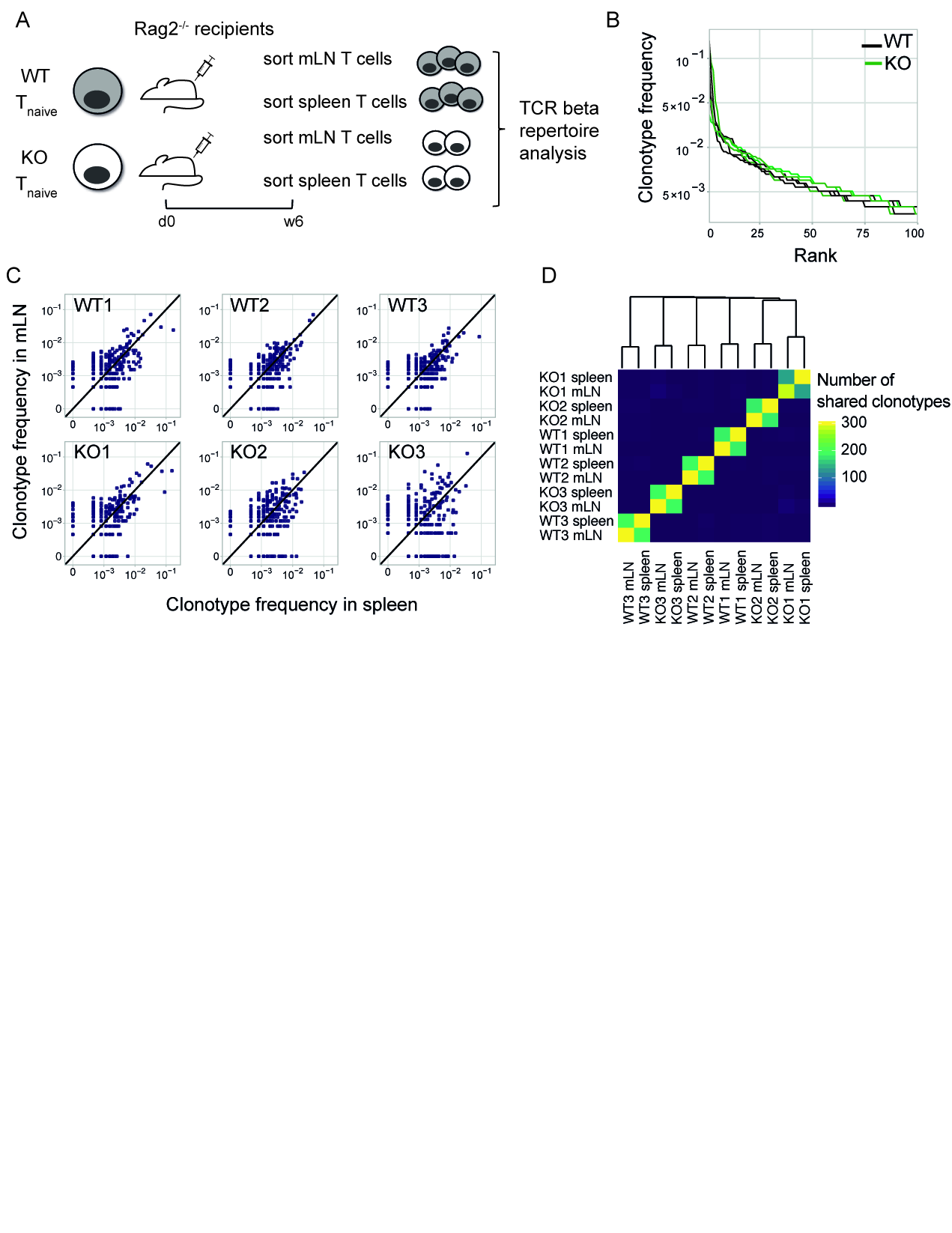
**

## Supplemental Figure 3. AMPK deficient T cells maintain homeostatic proliferation upon *in vivo* activation.

Panel A) shows the experimental set up of TCR beta repertoire analysis. Panel B) shows a rank-frequency (log-scale) distribution for the 100 most abundant WT and KO TCR beta clonotypes in the spleen. Panel C) shows frequencies of TCR beta clonotypes in the spleen (x-axis) vs. in mLN (y-axis) for each mouse. Diagonal line shows identity. Panel D) depicts the number of shared TCR beta nucleotide sequences between the 300 most abundant clonotypes from different organs (spleen vs. LN) and different mice. Dendrogram on top shows hierarchical clustering of samples based on the number of shared clonotypes. Data are obtained from one experiment with n=3 mice per group.
