# Supplementary material for: The energy sensor AMPK orchestrates metabolic and translational adaptation in expanding T helper cells
Source: FASEB J. 2021 Mar 14;35(4):e21217. doi: 10.1096/fj.202001763RR (PMC8252394; doi:10.1096/fj.202001763RR)
Supplement: Supplementary file 4 — Fig S4 [file FSB2-35-0-s004.docx]

# Supplemental Figure 4

**
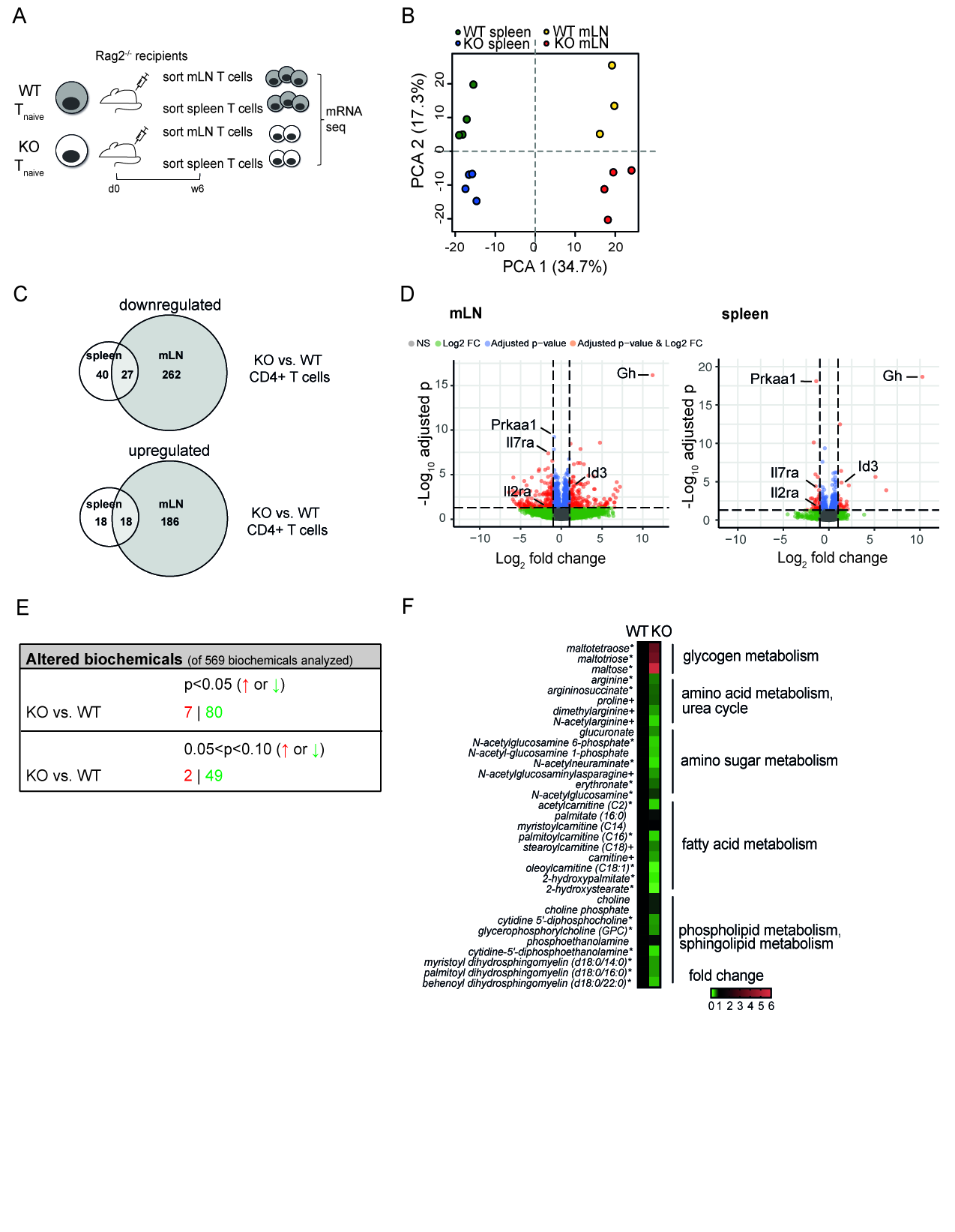
**

**Supplemental Figure 4. Gene expression profiles and metabolomic analysis of AMPK deficient T cells.**

A) shows the experimental set up of mRNA seq analysis. B) shows principal component analysis of WT and KO spleen and LN samples isolated 6 weeks after adoptive transfer. C) Venn diagrams depicting shared differential gene expression of WT and KO samples 6 weeks after transfer. D) Volcano plot showing differential gene expression (fold change vs. significance) of WT and KO mLN and spleen T cells. E) Summary of metabolomic analysis in activated WT and KO T cells. F) Metabolite levels of indicated biochemicals as determined in E). Data shown in A) – D) are obtained from one experiment with n=4 mice per groups. Data shown in E) and F) were obtained in 4 independent experiments with n=5-10 pooled mice per replicate per group. * *P*<0.05, + 0.05<*P*<0.10, paired student’s t test with Welch correction.
