# Supplementary material for: The energy sensor AMPK orchestrates metabolic and translational adaptation in expanding T helper cells
Source: FASEB J. 2021 Mar 14;35(4):e21217. doi: 10.1096/fj.202001763RR (PMC8252394; doi:10.1096/fj.202001763RR)
Supplement: Supplementary file 5 — Fig S5 [file FSB2-35-0-s001.docx]

# Supplemental Figure 5

**
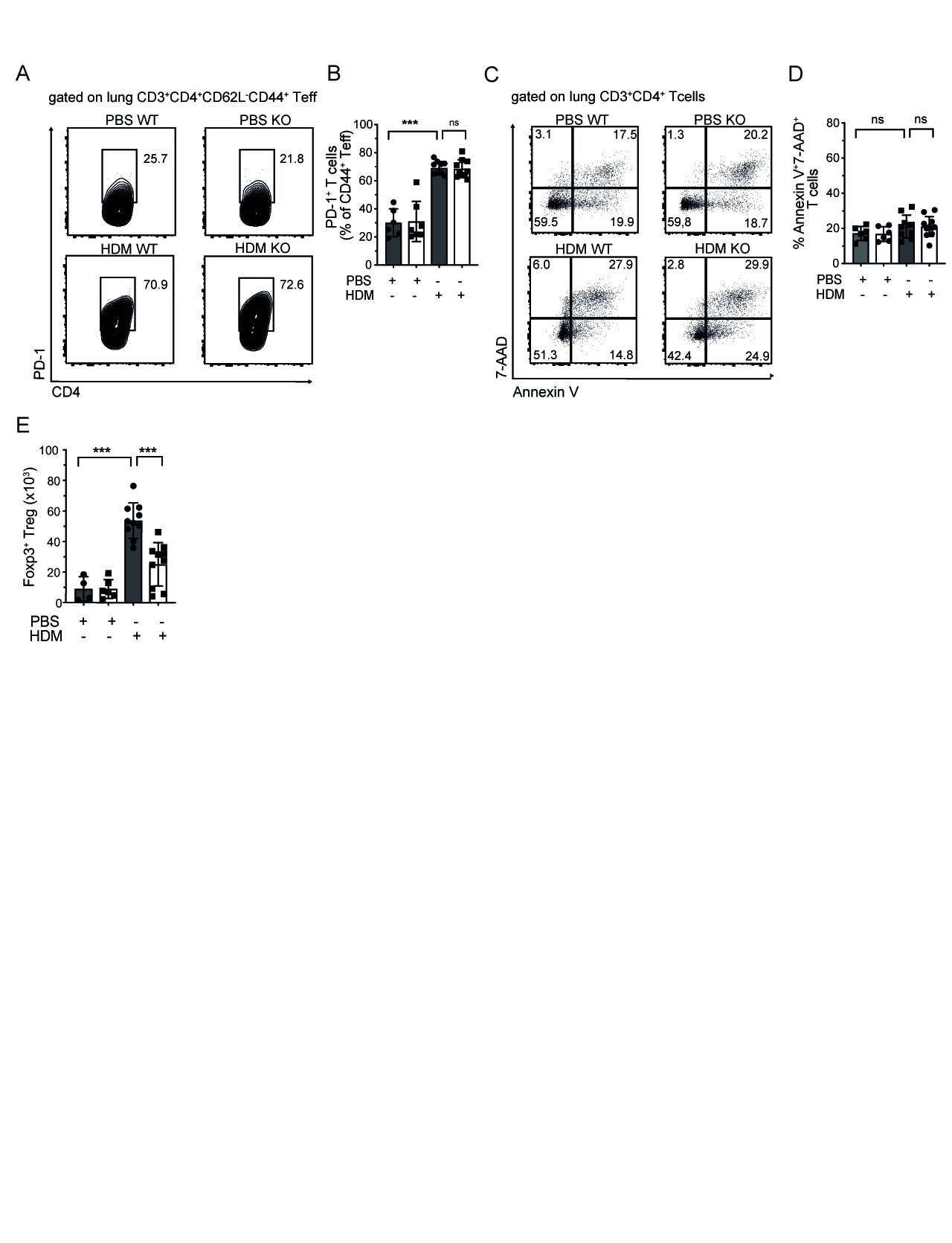
**

**Supplemental Figure 5. AMPK deficient T cells show no exhaustion phenotype or increased cell death upon *in vivo* HDM antigen exposure.**

A) Representative staining for PD-1 expression on CD44+CD62L- lung T effector cells. B) indicates the quantification of A). C) Representative Annexin-V vs. 7-AAD staining gated on lung CD3+CD4+ T cells at 14d after induction of allergic airway inflammation. D) indicates the quantification of C). E) Relative and absolute counts of lung Foxp3+ Treg 14d after induction of allergic airway inflammation. Data shown in A) and C) are representative of two independent experiments with 2-6 mice per group. Data shown in B), D) and E) indicate mean ± SD and are pooled data from two independent experiments with n=2-6 mice per group. * *P*<0.05, ** *P*<0.01, *** *P*<0.001, one-way ANOVA.
